# Inflammatory Signatures in MDS: The Missing Link Between Genetics, Microenvironment, and Therapy

**DOI:** 10.3390/cells15131137

**Published:** 2026-06-23

**Authors:** Adele Bottaro, Maria Elisa Nasso, Giuseppe Mirabile, Manlio Fazio, Alessandro Allegra

**Affiliations:** Hematology Unit, Department of Human Pathology in Adulthood and Childhood “Gaetano Barresi”, University of Messina, Via Consolare Valeria, 98125 Messina, Italy; adelebottarp15@gmail.com (A.B.); giuseppe.mirabile@polime.it (G.M.);

**Keywords:** myelodysplastic syndromes, inflammation, inflammaging, clonal hematopoiesis, bone marrow microenvironment, innate immunity, NLRP3 inflammasome, IRAK4–NFκB signaling, spliceosome mutations, *TP53* mutations

## Abstract

**Highlights:**

**What are the main findings?**

- Chronic inflammation and inflammaging act as active drivers of clonal selection, immune dysfunction, and bone marrow niche remodeling in myelodysplastic syndromes.
- Genetic lesions such as *TP53* and spliceosome mutations amplify inflammatory signaling, conferring clonal fitness and promoting disease evolution.

**What are the implications of the main findings?**

- Inflammatory signatures in peripheral blood provide accessible biomarkers for prognosis, risk stratification, and the prediction of therapeutic response in MDS.
- Integrating inflammatory profiling with genomic data supports the development of targeted therapies aimed at limiting inflammation-driven clonal evolution and restoring marrow homeostasis.

**Abstract:**

Myelodysplastic syndromes are clonal hematopoietic neoplasms in which ineffective hematopoiesis arises within the context of chronic inflammation and immune dysregulation. Growing evidence indicates that aging-associated inflammaging and inflammation-driven remodeling of the bone marrow microenvironment are not secondary phenomena, but active forces that shape clonal selection, lineage commitment, and disease evolution. This narrative review integrates recent insights from translational immunology, stem cell biology, multi-omics analyses, and clinical studies to examine the reciprocal interplay between inflammation and myelodysplastic syndrome pathogenesis. Chronic inflammatory stress imposes selective pressure on hematopoietic stem cells, favoring the expansion of mutation-bearing clones characteristic of clonal hematopoiesis and overt disease. As inflammation persists, immune dysfunction, together with stromal alterations, progressively reinforce ineffective hematopoiesis and clonal dominance. Genetic lesions, including TP53 and spliceosome mutations, further amplify inflammatory signaling and reshape the marrow niche, conferring clonal fitness and genomic instability. Clinically, readily accessible peripheral blood inflammatory indices reflect these biological processes and correlate with prognosis and therapeutic response. Collectively, these observations position inflammation as a unifying determinant of myelodysplastic syndrome initiation, progression, and treatment sensitivity. Integrating inflammatory signatures with genomic profiling may refine risk stratification and support the development of therapeutic strategies aimed at restoring marrow homeostasis and limiting inflammation-driven clonal evolution.

## 1. Introduction

Myelodysplastic neoplasms (MDS), formerly referred to as myelodysplastic syndromes, are a heterogeneous group of clonal hematopoietic disorders characterized by ineffective hematopoiesis, cytopenias, dysplasia of one or more myeloid lineages, and an increased risk of transformation to acute myeloid leukemia (AML) [1]. Traditionally conceptualized as diseases driven primarily by acquired genetic and epigenetic lesions, MDS are now recognized as conditions in which both somatic alterations and germline predisposition contribute to disease development, with inherited mutations representing a significant subset of adult-onset cases [2]. In fact, in addition to acquired somatic alterations, germline predisposition has emerged as an important contributor to MDS pathogenesis, particularly in adult-onset disease. Among these, mutations in *DDX41* represent one of the most frequent inherited lesions associated with myeloid neoplasms [3,4]. Beyond their role in genetic susceptibility, *DDX41* alterations are mechanistically linked to innate immune dysregulation, as loss of DDX41 function promotes the accumulation of nucleic acid structures and activation of the cGAS–STING inflammatory pathway, leading to enhanced production and expansion of hematopoietic stem and progenitor cells [5]. These findings support a direct biological connection between germline predisposition and inflammation-driven clonal hematopoiesis.

Over the last decade, the integration of genomic, immunological, and microenvironmental studies has substantially reshaped the understanding of MDS pathophysiology, revealing that inflammatory signals are not merely epiphenomena but active forces shaping clonal selection and disease evolution [6].

Aging represents the strongest epidemiological determinant of MDS incidence. Age-associated alterations in the hematopoietic stem cell (HSC) compartment—collectively referred to as immunosenescence—include reduced self-renewal capacity, skewing toward myeloid-biased differentiation, accumulation of somatic mutations, and impaired immune surveillance. These changes are closely linked to inflammaging, a state of chronic, low-grade, sterile inflammation driven by cytokines such as IL-1β, IL-6, TNF-α, and IFN-γ [7]. In this inflammatory milieu, normal hematopoiesis is progressively disadvantaged, while mutated HSC clones—particularly those harboring lesions that confer resistance to inflammatory stress (e.g., *TP53*, *TET2*, *ASXL1*)—acquire a selective fitness advantage [2,6]. Experimental and clinical evidence demonstrates that inflammatory cytokines exert differential effects on normal versus mutated hematopoietic stem cells (HSCs), creating a Darwinian selection pressure that favors clonal expansion [8].

This inflammatory framework is tightly intertwined with clonal hematopoiesis of indeterminate potential (CHIP), a prevalent age-related condition detectable in up to 10–20% of individuals over 70 years of age. CHIP is sustained by a bidirectional relationship with systemic inflammation: chronic inflammatory signaling promotes clonal expansion, while mutant clones—particularly those carrying *TET2* or *DNMT3A* mutations—actively reinforce inflammatory circuits within the hematopoietic and immune compartments. This self-perpetuating loop increases the likelihood of progression from CHIP to overt MDS [9].

Beyond HSC-intrinsic mechanisms, the bone marrow microenvironment plays a decisive role in shaping MDS biology. Aging and chronic inflammation remodel key niche components, including mesenchymal stromal cells, endothelial cells, macrophages, osteolineage cells, and adipocytes. Stromal elements from MDS patients display increased NF-κB activation, impaired hematopoietic support, and enhanced production of inflammatory mediators, collectively contributing to ineffective hematopoiesis and niche dysfunction [10]. In parallel, dysplastic clones actively reshape the immune landscape through immune evasion and immunosuppressive mechanisms, further reinforcing clonal dominance [11].

From a clinical standpoint, inflammation correlates closely with disease severity and prognosis. Distinct inflammatory profiles differentiate lower- and higher-risk MDS, and circulating inflammatory markers have been repeatedly associated with survival, transfusion dependence, leukemic progression, and response to hypomethylating agents [12]. These observations underscore the clinical relevance of inflammatory processes beyond their mechanistic role.

Importantly, rather than considering these processes as independent layers of disease biology, we propose that they should be interpreted within a unified framework in which inflammation represents the central organizing force linking genetic alterations, immune dysregulation, and microenvironmental remodeling. In this perspective, MDS evolution reflects a dynamic and self-reinforcing system in which inflammatory signaling, clonal selection, and niche adaptation continuously interact and reshape each other.

Building on these concepts, this review summarizes current evidence supporting inflammation as a central organizing principle in MDS pathogenesis. The following sections examine how aging-related inflammatory changes promote clonal selection, how innate and adaptive immune dysfunction and niche alterations reinforce ineffective hematopoiesis, and how specific genetic lesions intersect with inflammatory signaling to shape disease heterogeneity. Finally, the review discusses the prognostic and therapeutic implications of inflammatory signatures, highlighting emerging strategies aimed at modulating inflammation to improve risk stratification and treatment outcomes [13].

During the preparation of this manuscript, Microsoft 365 Copilot (GPT-5 chat model) was used solely as an auxiliary tool for language refinement, figure conceptualization, and organizational support. The authors have reviewed and edited the output and take full responsibility for the content of this publication.

### A Unifying Inflammation-Centered Model of MDS Evolution

Rather than representing parallel and partially independent pathogenic processes, inflammation, clonal hematopoiesis, immune dysregulation, and microenvironmental remodeling can be interpreted as components of a tightly interconnected and dynamic system that drives MDS evolution. We propose a unifying conceptual framework in which inflammatory signaling acts as the primary organizing force that integrates these domains across temporal and biological scales.

Within this model, inflammaging establishes the initial permissive landscape, characterized by chronic cytokine exposure, oxidative stress, and impaired resolution pathways. This environment exerts a selective pressure on HSCs, favoring the emergence and expansion of clones harboring mutations that confer resistance to inflammatory stress. The transition from physiological aging to clonal hematopoiesis of indeterminate potential (CHIP) and subsequently to clonal cytopenia of undetermined significance (CCUS) reflects not merely genetic accumulation, but progressive adaptation to an inflammatory ecosystem.

As clonal hematopoiesis emerges, the relationship with inflammation becomes bidirectional. Mutated clones—particularly those carrying lesions in epigenetic regulators, spliceosome components, or TP53—actively amplify inflammatory circuits through altered transcriptional programs, aberrant RNA species, and enhanced innate immune signaling. This establishes a self-reinforcing feedback loop in which inflammation promotes clonal expansion, and mutant clones further sustain inflammation.

Concurrently, the bone marrow microenvironment undergoes inflammation-driven remodeling, transitioning from a homeostatic niche to a dysfunctional and permissive ecosystem. Stromal cells, endothelial elements, and immune populations respond to chronic inflammatory stimuli by adopting pro-inflammatory and immunosuppressive phenotypes, thereby reinforcing clonal dominance and suppressing normal hematopoiesis. This process of “niche reprogramming” represents a central amplifier of disease progression.

Importantly, this framework also accommodates the stage-dependent evolution of immune responses, with early pro-inflammatory and immune-activated states progressively shifting toward immune exhaustion and tolerance in advanced disease. These transitions contribute to both phenotypic heterogeneity and therapeutic response variability.

From a translational perspective, this integrated model suggests that MDS should be viewed not only as a genetically defined malignancy, but as a system-level disorder of dysregulated inflammatory signaling and ecosystem imbalance. Consequently, inflammatory signatures—when integrated with genomic and clinical data—may provide a biologically grounded approach to risk stratification and therapeutic targeting.

Together, this inflammation-centered framework provides a coherent interpretation of MDS pathogenesis, bridging molecular lesions, immune dysfunction, and microenvironmental remodeling into a unified model of disease initiation, progression, and treatment response.

## 2. From Physiological Aging to Pathological Clonal Expansion: The Inflammaging Connection

Inflammaging, a defining feature of biological aging, reflects a persistent low-grade inflammatory state that emerges from cumulative cellular senescence, mitochondrial dysfunction, genomic instability, and impaired resolution of inflammatory responses [14].

Senescent cells accumulate with age and adopt a senescence-associated secretory phenotype (SASP) releasing IL-6, IL-8, IL-1β, and TNF-α. These mediators reshape the bone marrow milieu by activating the NF-κB, p38 MAPK, and JAK/STAT pathways in HSCs, driving myeloid-biased differentiation, loss of quiescence, and diminished long-term reconstitution capacity—changes that phenocopy early MDS features. In parallel, aging-associated mitochondrial dysfunction increases reactive oxygen species (ROS), which promote DNA damage and activate inflammasomes such as NLRP3, perpetuating inflammatory cycling.

In addition to mitochondrial dysfunction, aging-associated inflammation exerts direct effects on hematopoietic stem cells through cytokine-mediated signaling, including IL-1, TNF-α, and interferons, which drive a loss of quiescence, increased proliferative stress, and skewed differentiation. These processes create a selective environment favoring clones harboring mutations that confer resistance to inflammatory stress.

Chronic inflammatory signaling also promotes replication stress and DNA damage accumulation in HSCs while simultaneously reshaping the bone marrow niche toward a pro-inflammatory and less supportive state. Together, these mechanisms favor the emergence and expansion of mutated clones, linking physiological aging to clonal hematopoiesis and ultimately to MDS [7,15,16,17].

Metabolic reprogramming toward glycolysis further limits the regenerative fitness of aged HSCs. Moreover, inflammaging is characterized by the impaired production of specialized pro-resolving mediators (SPMs), such as resolvins and lipoxins, which are normally required to terminate inflammatory responses. Failure of resolution mechanisms promotes the persistence of inflammatory stress, a key permissive step for the evolution from age-related hematopoietic dysfunction toward clonal expansion and eventual MDS [18,19,20]. These observations support the concept that inflammaging is not merely a background condition, but acts as a selective ecological pressure that actively shapes hematopoietic fitness and initiates the continuum from physiological aging to clonal hematopoiesis and overt malignancy.

## 3. Inflammation-Driven Clonal Hematopoiesis as a Precursor to MDS

While inflammaging establishes a permissive biological background, clonal hematopoiesis (CH)—particularly CHIP—emerges as the first measurable inflection point linking aging, inflammation, and the development of MDS.

CHIP arises when HSCs acquire mutations—most commonly in *DNMT3A*, *TET2*, *ASXL1*, *TP53*, *PPM1D*, or *JAK2*—that confer a competitive advantage without causing overt cytopenias. Its prevalence exceeds 10% in older individuals [21,22].

Chronic inflammation functions as a key driver of clonal selection, whereas mutated clones display enhanced inflammatory fitness. For example, *TET2*-mutant HSCs show attenuated STAT3 signaling in response to IL-6, *DNMT3A*-mutant cells resist IFN-γ-induced exhaustion, and ASXL1 mutations upregulate inflammatory transcriptional programs that support clonal persistence. This clone-driven inflammatory amplification contributes to the systemic manifestations observed in CHIP carriers, including elevated cardiovascular risk and autoimmune phenomena [23,24,25].

Persistent inflammatory stress accelerates progression from CHIP to CCUS and ultimately MDS. Chronic cytokine exposure induces replication stress, mitochondrial damage, and impaired DNA repair, promoting the acquisition of secondary mutations. Moreover, preexisting age-related niche alterations—reduced osteoblastic support, dysfunctional MSC immunoregulation, and increased adipogenesis—provide a selective advantage for mutated clones in the inflamed marrow environment [26,27,28]. Furthermore, pattern recognition receptors (PRRs) represent a fundamental interface between inflammatory signals and clonal hematopoiesis, as they detect pathogen-associated and damage-associated molecular patterns, including nucleic acids derived from endogenous retroelements. Their activation—through sensors such as Toll-like receptors, RIG-I–like receptors, and the cGAS–STING pathway—triggers downstream inflammatory programs, including NF-κB and interferon signaling, thereby promoting hematopoietic stem cell stress, clonal selection, and progression along the CHIP–CCUS–MDS continuum.

**Figure 1:**
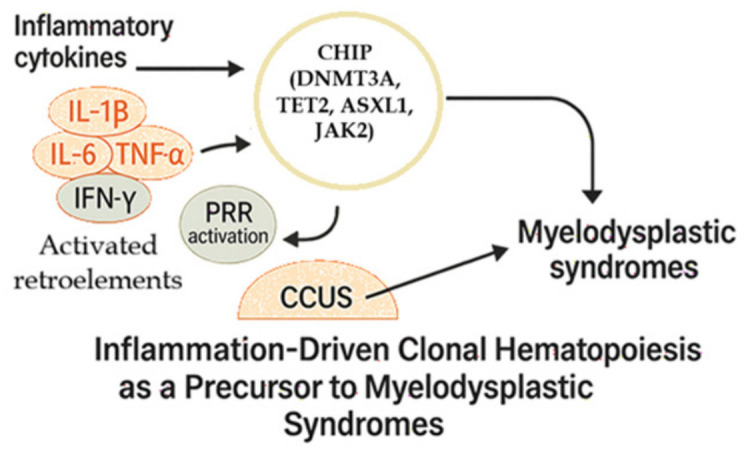
Chronic inflammatory cytokines and activation of pattern-recognition receptors (PRRs), triggered in part by retroelement-derived nucleic acids, promote the selective expansion of mutated hematopoietic clones associated with clonal hematopoiesis of indeterminate potential (CHIP), facilitating progression to clonal cytopenia of undetermined significance (CCUS) and ultimately to MDS.

Finally, CHIP-associated mutations disrupt innate immune sensing pathways driving sustained interferon signaling, HSC exhaustion, and clonal skewing, fostering progression toward overt MDS [29,30] (Figure 1).

**Figure 2:**
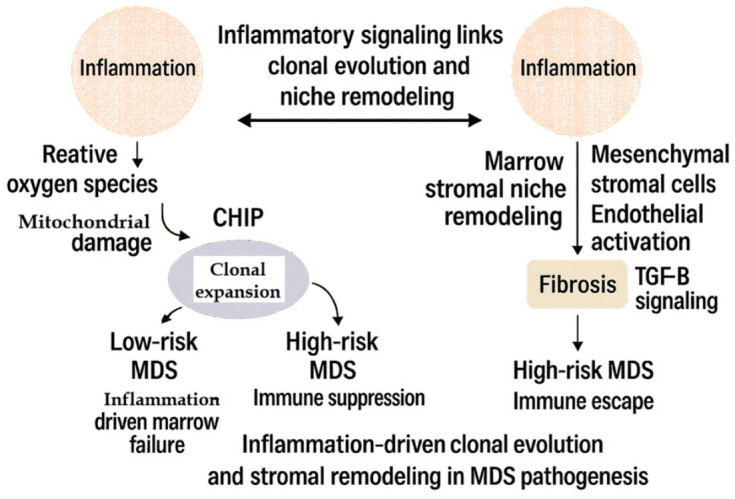
Inflammation-driven clonal evolution and stromal remodeling in MDS. Chronic inflammatory stress promotes hematopoietic stem cell dysfunction and the emergence of clonal hematopoiesis (CHIP), which expands through self-reinforcing inflammatory feedback and evolves toward low- or high-risk MDS. In parallel, persistent inflammation remodels the bone marrow microenvironment through the activation of mesenchymal stromal cells, endothelial dysfunction, and adipocyte-mediated signaling, ultimately leading to TGF-β-driven fibrosis. These interconnected processes establish a bidirectional loop that sustains disease progression.

Together, these mechanisms illustrate how inflammation functions simultaneously as a trigger, amplifier, and accelerator of clonal evolution. Targeting inflammatory circuits or senescent-cell programs represents a promising therapeutic avenue to interrupt the continuum from CHIP to MDS (Figure 2).

While inflammation has long been recognized as a key driver of clonal hematopoiesis, emerging evidence indicates that this relationship is bidirectional. Mutant hematopoietic clones associated with CH not only expand under inflammatory pressure but can also actively reshape the inflammatory milieu. Indeed, clones harboring mutations in genes such as *TET2* or *DNMT3A* have been shown to promote the exaggerated production of pro-inflammatory cytokines, thereby amplifying innate immune activation and establishing a self-reinforcing inflammatory loop.

In parallel, early alterations in adaptive immunity contribute to this process. T-cell compartments in the context of CH may exhibit functional dysregulation, including skewing toward exhausted or regulatory phenotypes, reduced cytotoxic activity, and impaired immune surveillance. These changes may facilitate the persistence and expansion of mutant clones. Moreover, the inflammatory environment associated with CH impacts the bone marrow stromal niche. Mesenchymal stromal cells and other niche components respond to chronic inflammatory signaling by altering their transcriptional programs and cytokine production, leading to a microenvironment that favors clonal dominance while impairing normal hematopoiesis. Together, these mechanisms support a model in which CH, immune dysregulation, and microenvironmental remodeling are tightly interconnected processes that precede and predispose to MDS development [31].

## 4. Innate Immune Circuits Driving MDS

Innate immune circuits refer to interconnected signaling pathways that regulate the recognition of danger signals and the initiation of inflammatory responses through pattern-recognition receptors, cytokine networks, and downstream transcriptional programs such as *NF-κB* activation. Under physiological conditions, these circuits preserve tissue homeostasis, regulate hematopoietic stem cell function, and coordinate effective immune responses to stress and infection. In MDS, however, these pathways become chronically activated and dysregulated, contributing to ineffective hematopoiesis and clonal selection [32].

### 4.1. Physiological Role of Innate Immune Circuits

Under physiological conditions, innate immune circuits regulate the recognition of danger-associated and pathogen-associated signals through pattern-recognition receptors such as Toll-like receptors and inflammasome components. These pathways coordinate rapid inflammatory responses via cytokine production and the activation of transcriptional programs including NF-κB signaling. In the bone marrow, innate immune signaling plays a critical role in maintaining hematopoietic homeostasis by regulating hematopoietic stem cell quiescence, proliferation, and differentiation in response to stress and infection. Tight regulation of these pathways is essential to prevent excessive inflammation and preserve effective hematopoiesis.

### 4.2. Innate Immune Dysregulation in MDS

Innate immunity is profoundly dysregulated in MDS, and abnormalities in pattern-recognition pathways, cytokine production, and monocyte/macrophage function contribute to both ineffective hematopoiesis and clonal advantage. Among all immune compartments, the innate arm exerts the earliest and most persistent influence on HSC behavior and marrow homeostasis in MDS [32].

Among the innate immune alterations, aberrant activation of Toll-like receptor signaling—particularly the MyD88–IRAK4 axis—represents one of the earliest and most influential pathogenic drivers in MDS.

In fact, a central pathogenic role is attributed to Toll-like receptor (TLR) signaling, especially TLR2, TLR4, and TLR9, which are frequently overexpressed in MDS progenitors. Activation of these receptors engages the MyD88 adaptor, leading to the downstream phosphorylation of IRAK1 and IRAK4. This culminates in NF-κB nuclear translocation and the transcription of inflammatory mediators. In many MDS subtypes, particularly those with *SRSF2* or *U2AF1* mutations, aberrant splicing produces constitutively active IRAK4 isoforms, which sustain NF-κB activity independently of ligand stimulation. This results in the chronic production of IL-8, IL-6, TNF-α, and GM-CSF, fostering a microenvironment hostile to normal hematopoiesis yet permissive for dysplastic progenitors. Preclinical studies demonstrate that inhibiting IRAK4 in splicing-mutated MDS restores normal erythroid differentiation and reduces inflammatory gene expression, highlighting a mechanistic convergence between innate immunity and mutational landscapes [33,34,35].

Monocytes and macrophages in MDS exhibit dysfunctional polarization and defective efferocytosis. Instead of adopting pro-resolving M2 phenotypes, MDS macrophages skew toward M1-like inflammatory states and demonstrate impaired clearance of dead cells. This inefficiency contributes to the accumulation of apoptotic bodies and necrotic debris within the marrow, amplifying inflammatory signaling through DAMP recognition. Furthermore, macrophage-derived S100A9 engages TLR4 and CD33 on HSCs and progenitors, activating suppressive circuits that impair erythroid differentiation and promote oxidative stress. This pathway is particularly active in del(5q) MDS, where haploinsufficiency of RPS14 increases S100A9 sensitivity [30,31,32].

Monocytes and macrophages in MDS show a dysregulated and plastic polarization state rather than a fixed M1 phenotype. Although inflammatory activation may characterize early disease, multiple studies have demonstrated a reduced M1/M2 ratio and expansion of M2-like macrophages in higher-risk MDS, accompanied by diminished pro-inflammatory function and enhanced support of clonal hematopoiesis. These findings indicate that macrophage polarization in MDS is dynamic, stage-dependent, and contributes to disease progression through immunosuppressive and tumor-supportive mechanisms [36,37].

Dysregulation of natural killer (NK) cells and dendritic cells (DCs) also characterizes innate immune dysfunction in MDS. NK cells display a reduced expression of cytotoxic mediators (perforin, granzyme B), decreased IFNγ output, and impaired recognition of dysplastic clones. Dendritic cells show functional immaturity, altered antigen presentation, and TLR hyporesponsiveness. Importantly, beyond these functional abnormalities, dendritic cells in MDS may derive from the malignant hematopoietic clone and harbor the same genetic alterations observed in MDS blasts, indicating that their dysfunction is at least partially driven by cell-intrinsic genetic defects in addition to microenvironmental influences [38,39,40].

Innate immune dysregulation also shapes the stromal niche. Mesenchymal stromal cells activated via TLR2/TLR4 pathways shift toward the production of IL-6, TGF-β, and prostaglandin E2, which inhibit erythropoiesis and promote fibrosis. Endothelial cells exposed to chronic inflammatory stimuli upregulate adhesion molecules (VCAM-1, ICAM-1), enhancing the retention of inflammatory myeloid cells. Adipocytes release fatty acids that induce oxidative stress and inflammasome activation in adjacent progenitors [34,41,42,43,44].

### 4.3. Clinical Implications: Outcomes and Therapeutic Response

Finally, innate immune activation contributes to response heterogeneity to hypomethylating agents (HMAs). Elevated levels of IL-8 or NF-κB activity predict poorer response to azacitidine, whereas patients with low baseline inflammatory gene expression experience more durable hematologic improvement. These insights underscore the profound prognostic significance of innate immunity in MDS and point toward therapeutic targets—such as IRAK4 inhibitors, NLRP3 inflammasome blockers, or IL-1β antagonists—that are currently under active clinical development [33,45,46,47,48].

In parallel, inflammasome activation constitutes a second core driver of innate immune dysregulation, linking inflammatory signaling to intramedullary cell death and ineffective hematopoiesis.

Dysplastic hematopoietic progenitors exhibit increased activation of the caspase-1 inflammasome complex, leading to the overproduction of IL-18. This cytokine has multiple pathogenic effects: they promote pyroptotic cell death, amplify inflammatory signaling via autocrine and paracrine loops, and impair erythroid maturation [49,50]. Pyroptosis, characterized by gasdermin D-mediated membrane pore formation, contributes to the excessive intramedullary apoptosis that typifies lower-risk MDS. The resulting release of DAMPs, including ATP, HMGB1, and mitochondrial DNA, serves as a secondary stimulus that further activates innate immune pathways and perpetuates marrow injury [51].

Acting primarily as an inflammatory amplifier rather than an initiating event, the S100A8/A9 alarmin axis sustains innate immune activation once dysregulation is established, reinforcing inflammatory stress and suppressing normal hematopoiesis. These calcium-binding proteins, produced by myeloid-derived cells, bind TLR4 and CD33, driving a self-perpetuating inflammatory cycle. In del(5q) MDS, S100A8/A9 expression is markedly increased, contributing to erythroid apoptosis, mitochondrial dysfunction, and enhanced oxidative stress. These factors suppress normal hematopoiesis while conferring a survival advantage to dysplastic clones [52,53,54].

Innate immune dysfunction extends to dendritic cells, which are both quantitatively and functionally abnormal in MDS. Dendritic cells show diminished antigen-presentation ability, downregulated costimulatory molecules, and impaired capacity to activate naive T cells. These abnormalities contribute to both chronic inflammation and defective immune surveillance [40,55].

Sustained activation of innate immune pathways translates into functional impairment and the exhaustion of innate immune effector cells, including neutrophils. Neutrophils in MDS display impaired chemotaxis, defective phagocytosis, and increased formation of neutrophil extracellular traps (NETs) [56]. NETs contain oxidized DNA and proteases that further activate innate immune receptors and stimulate inflammasome signaling in the marrow [57,58]. These functional abnormalities are driven by multiple intersecting pathogenic mechanisms. Chronic inflammatory signaling and persistent exposure to cytokines such as IL6 and TNFα promote neutrophil activation but also induce functional exhaustion. Increased oxidative stress and mitochondrial dysfunction enhance ROS production, which is a key trigger of NET formation. In parallel, activation of innate immune pathways—including TLR signaling and inflammasome priming—further amplifies NETosis through NFκB-driven transcriptional programs. Moreover, dysplastic hematopoiesis and clonal alterations contribute to aberrant neutrophil differentiation, resulting in the expansion of dysfunctional neutrophil subsets characterized by impaired antimicrobial activity but enhanced pro-inflammatory output. Collectively, these mechanisms link neutrophil dysfunction and excessive NET formation to the chronic inflammatory microenvironment that sustains MDS progression [57,58].

**Figure 3:**
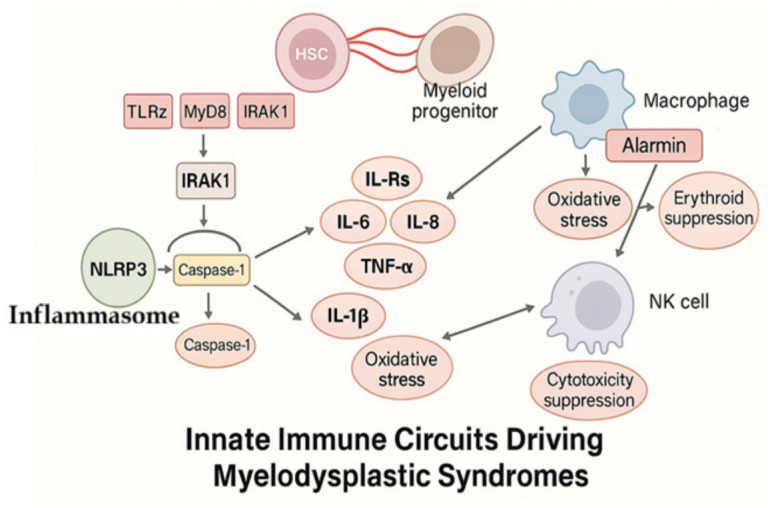
Activation of TLR–MyD88–IRAK1 signaling and the NLRP3 inflammasome leads to caspase-1-mediated maturation of IL-1β and the increased secretion of pro-inflammatory cytokines (IL-6, IL-8, TNF-α). These pathways induce oxidative stress, impair myeloid and erythroid differentiation, and suppress macrophage and NK cell functions, collectively contributing to ineffective hematopoiesis and MDS development. **Abbreviations:** TLR: Toll-like receptor; MyD88: Myeloid differentiation primary response 88; IRAK1: Interleukin-1 receptor–associated kinase 1; NLRP3: NACHT, LRR, and PYD domains-containing protein 3; HSC: Hematopoietic stem cell.

Innate immune abnormalities also correlate with clinical outcomes. Elevated IL-8 levels predict poor response to hypomethylating agents, while high NF-κB activity in progenitors correlates with progression to AML. Altogether, innate immunity in MDS is characterized by persistent, maladaptive activation of inflammatory pathways that contribute to ineffective hematopoiesis, clonal dominance, and disease progression [45] (Figure 3, Table 1).

## 5. Adaptive Immunity at the Crossroads of Clonal Evolution in MDS

Adaptive immunity refers to antigen-specific immune responses mediated by T and B lymphocytes, characterized by immunological memory and the precise recognition of non-self-antigens. Under physiological conditions, adaptive immune responses ensure the effective elimination of pathogens and malignant cells while maintaining immune tolerance and preventing autoimmunity. In the bone marrow, adaptive immunity contributes to immune surveillance and the regulation of hematopoiesis through coordinated interactions between T-cell subsets, B cells, and antigen-presenting cells [59].

Adaptive immune dysfunction in MDS follows a dynamic trajectory that evolves over the course of disease. Early stages are often characterized by immune activation and autoimmune phenomena, whereas disease progression is associated with progressive immune exhaustion and suppression. This temporal shift profoundly influences clonal selection, disease phenotype, and therapeutic responsiveness.

Adaptive immunity in MDS is profoundly remodeled and contributes both to early disease phenotypes (autoimmune manifestations, cytopenias from immune-mediated destruction) and to later disease progression (immune exhaustion and immune evasion). The adaptive immune system shapes the clonal architecture of MDS and affects therapeutic response [11,60].

A hallmark of adaptive immune dysfunction in MDS is T cell exhaustion. CD8^+^ cytotoxic T lymphocytes show persistent overexpression of inhibitory receptors—including PD-1, TIM-3, LAG-3, and TIGIT—and diminished production of IFN-γ and TNF-α. This results in impaired cytotoxicity and ineffective clearance of malignant or pre-malignant clones. Persistent antigen exposure from dysplastic cells and DAMP-induced signaling drives the expansion of exhausted T cell subsets. The degree of T cell exhaustion correlates with higher blast percentages and worse overall survival [11,47,61].

At the molecular level, this exhausted phenotype is driven in part by direct interactions between immune checkpoint ligands expressed on MDS blasts and inhibitory receptors on T cells. In particular, programmed death-ligand 1 and 2 (PD-L1/PD-L2) expressed on dysplastic progenitors engage PD-1 on CD8^+^ T cells, leading to the inhibition of T-cell receptor signaling, reduced cytokine production, and impaired cytotoxic function. Similarly, Galectin-9 expressed by MDS cells binds to T-cell immunoglobulin and mucin-domain containing-3 (TIM-3), further promoting T-cell exhaustion and apoptosis. These checkpoint interactions establish a profoundly immunosuppressive microenvironment, enabling malignant clones to evade immune surveillance and progressively expand [11,47,61].

Simultaneously, many MDS patients exhibit increased regulatory T cells (Tregs). These Tregs overexpress FOXP3, CTLA-4, and ICOS, enhancing their suppressive activity. By inhibiting cytotoxic T cell and NK cell responses, Tregs create an immunosuppressive niche that favors malignant clone survival. Elevated Treg numbers are especially prominent in high-risk MDS and are linked to mutations in genes such as *ASXL1* and *TP53*, which may further skew immune tolerance [62,63].

Helper T-cell subsets are also altered. In certain lower-risk MDS patients, Th17 cells and IL-17 levels are elevated, promoting inflammatory apoptosis and contributing to marrow failure. In contrast, higher-risk cases often exhibit a shift toward Th2 polarization and reduced Th1 cytotoxic responses, paralleling the transition from autoimmunity toward immunosuppression [59].

B-cell immunity is also impaired. B-cell lymphopenia is common and reflects diminished lymphoid output from the HSC pool. Clonal restriction of the B-cell receptor repertoire, reduced somatic hypermutation, and impaired class-switch recombination compromise humoral immunity and increase infection susceptibility. These abnormalities also reduce anti-leukemic antibody responses [59].

Natural killer cells, bridging innate and adaptive immunity, display reduced cytotoxicity, decreased expression of activating receptors, and increased inhibitory signaling. NK cell dysfunction contributes significantly to immune escape by dysplastic clones [38,64].

Adaptive immune alterations contribute to clinical heterogeneity. Early MDS frequently presents with autoimmune phenomena—arthritis, cutaneous vasculitis, autoimmune cytopenias—reflecting hyperactive yet dysregulated adaptive immunity. Advanced MDS presents instead with dominant immune exhaustion, aiding in disease progression [65].

Adaptive immunity also modulates the response to therapy. Patients with lower exhaustion markers respond better to hypomethylating agents, while highly exhausted T cell profiles predict poor outcomes. Emerging therapies such as checkpoint inhibitors, anti-CD47 antibodies, and T cell–based immunomodulators are being investigated to restore adaptive immune function [66].

Thus, adaptive immunity in MDS evolves from early hyperactivation to late-stage exhaustion, shaping disease phenotype, clonal architecture, and therapeutic responses.

## 6. The Inflamed Niche: How Bone Marrow Ecosystems Drive MDS Evolution

Under physiological conditions, the bone marrow microenvironment is a highly organized and dynamic niche composed of mesenchymal stromal cells, endothelial cells, osteolineage cells, and immune populations that collectively regulate hematopoietic stem cell quiescence, self-renewal, and differentiation. These interactions ensure balanced hematopoiesis and immune homeostasis [67,68].

With aging, this tightly regulated system undergoes progressive alterations driven by chronic low-grade inflammation (inflammaging). Mesenchymal stromal cells acquire a senescent phenotype characterized by reduced proliferative capacity, mitochondrial dysfunction, genomic instability, and activation of NFκB. These senescent MSCs develop a senescence-associated secretory phenotype (SASP), releasing inflammatory mediators such as TGFβ, CXCL12, and CCL2, which disrupt normal hematopoietic support, impair erythroid and megakaryocytic differentiation, and promote myeloid skewing [69,70].

In parallel, chronic inflammatory signaling progressively destabilizes the bone marrow niche, creating a permissive environment for clonal selection. Within this context, mutated hematopoietic clones actively remodel the microenvironment in a process known as niche hijacking. Mutant progenitors release cytokines, exosomes, and mitochondrial components that reprogram stromal cells toward pro-inflammatory phenotypes. For example, SF3B1-mutant clones alter mitochondrial metabolism and impair osteoblast differentiation, while TP53-mutant clones induce DNA damage responses and inflammatory gene expression in stromal elements [71,72].

These combined effects of aging, inflammation, and clonal hematopoiesis progressively alter the structural and functional components of the bone marrow niche. The vascular compartment is disrupted, as endothelial cells exposed to chronic inflammatory stimuli upregulate adhesion molecules such as E-selectin, VCAM1, and ICAM1, promoting the abnormal retention of inflammatory cells and enhancing angiogenesis. Persistent oxidative stress further impairs vascular integrity and destabilizes HSC quiescence [73,74,75,76].

Another hallmark feature is the expansion of bone marrow adiposity. Adipocytes accumulate with aging and release inflammatory mediators, including IL6, leptin, and fatty acids, which suppress erythropoiesis, increase reactive oxygen species (ROS), and impair HSC function. These adipocyte-rich niches further contribute to inflammasome activation and reinforce the inflammatory microenvironment [77,78,79,80].

In addition, chronic exposure to TGFβ and inflammatory cytokines promotes fibroblast activation and extracellular matrix deposition, leading to bone marrow fibrosis. This structural remodeling disrupts normal stromal–hematopoietic interactions, alters oxygen and nutrient gradients, and displaces HSCs from their physiological niches, favoring proliferation over quiescence. Clinically, fibrosis correlates with adverse prognosis and increased transfusion requirements [81,82,83].

**Figure 4:**
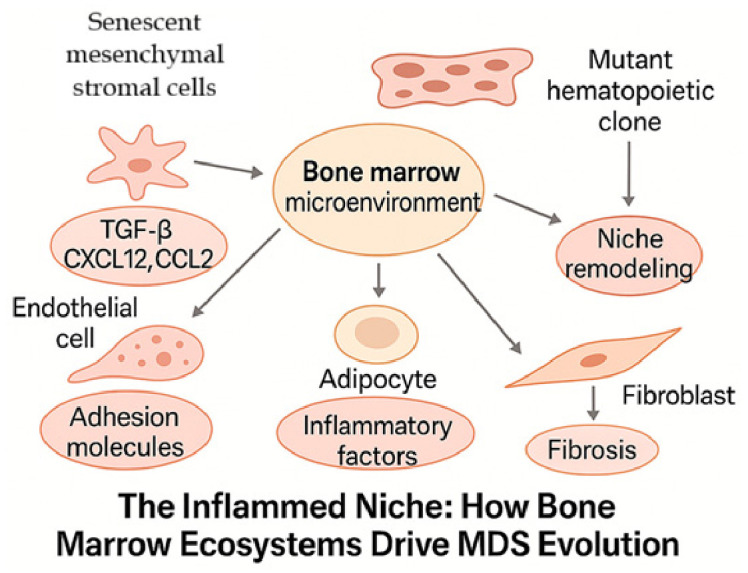
Senescent mesenchymal stromal cells, dysfunctional endothelial cells, adipocytes, fibroblasts, and mutated hematopoietic clones contribute to an inflamed and structurally altered bone marrow microenvironment. Through the secretion of inflammatory mediators (TGF-β, CXCL12, CCL2), expression of adhesion molecules, release of adipocyte-derived inflammatory factors, and stromal remodeling leading to fibrosis, the niche becomes supportive of clonal expansion and accelerates progression toward myelodysplastic syndrome.

Collectively, these processes transform the bone marrow from a homeostatic regulatory environment into a chronically inflamed and functionally impaired niche. This remodeled microenvironment supports clonal expansion, suppresses normal hematopoiesis, and accelerates disease progression in MDS (Figure 4).

## 7. Inflammatory Selection and Genomic Destabilization in TP53-Mutant MDS

Among the various genetic lesions implicated in MDS, *TP53* mutations represent the most clinically ominous subset. *TP53*-mutant MDS is strongly associated with complex karyotypes, chromosomal instability, resistance to hypomethylating agents, rapid progression to AML, and extremely poor survival [84]. Recent high-resolution studies have revealed an underappreciated dimension of *TP53* biology: an intimate and bidirectional interaction between inflammatory signaling and clonal evolution, whereby chronic inflammation not only selects for *TP53*-mutant HSCs but also amplifies their genomic instability and leukemogenic potential [85].

### Inflammation as a Selective Driver of TP53-Mutant Fitness

Under physiological conditions, HSCs rely on p53 to maintain genomic integrity, promote DNA repair, and preserve quiescence [86]. However, in inflammatory microenvironments rich in IL-1β, TNF-α, and IFN-γ, wild-type HSCs experience oxidative stress, replication stress, and mitochondrial dysfunction, leading to apoptosis or senescence. In contrast, TP53-mutant HSCs become inappropriately resistant to these stresses. Murine chimera experiments demonstrate that chronic administration of poly(I:C) or lipopolysaccharide (LPS)—mimicking viral and bacterial inflammation—selectively expands TP53-mutant HSCs at the expense of normal ones. After repeated inflammatory hits, mutant clones occupy an increasing fraction of the HSC pool, reflecting a profound fitness advantage in inflammatory settings [87].

Chronic inflammation promotes oxidative and replicative stress, leading to the accumulation of DNA damage and defective checkpoint activation. In *TP53*-mutant cells, impaired cell cycle arrest and DNA repair responses allow for survival and expansion despite genotoxic stress, conferring a strong selective advantage within an inflammatory microenvironment.

It was demonstrated that inflammation accelerates genomic instability in TP53-mutant clones. As reported, whereas wild-type *p53* enables cells to halt proliferation and repair damage, *TP53*-mutant cells fail to mount appropriate responses to genotoxic and oxidative stress. Under repeated inflammatory stimulation, these mutant HSCs accumulate large numbers of chromosomal aberrations, copy-number alterations, and driver mutations. Single-cell multiomic studies reveal that *TP53*-mutant clones show the pre-activation of inflammatory gene programs (e.g., TNFA signaling, IFN pathways, NF-κB targets) even before clinical transformation. This indicates that inflammation is not only permissive but actively contributes to the acquisition of additional leukemogenic events [88].

In addition, mutant *p53* actively shapes the inflammatory microenvironment by promoting NF-κB signaling and pro-inflammatory cytokine production, thereby reinforcing a self-sustaining loop between clonal expansion and inflammation [85].

Longitudinal studies of *TP53*-mutated patients show distinct patterns of clonal evolution:

- Bi-allelic *TP53* inactivation,
- Hemizygous mutations with progressive allelic imbalance,
- Parallel evolution among multiple *TP53*-hit subclones.

Remarkably, inflammatory stimulation preferentially promotes bi-allelic *TP53* disruption, the genomic configuration most strongly associated with leukemic transformation. TNF-α and IL-1β impair DNA double-strand break repair through p53-dependent and independent pathways, accelerating the transition from monoallelic to multi-hit TP53 states [89,90].

Moreover, *TP53*-mutant HSCs reshape the niche by secreting inflammatory mediators and mitochondrial DNA fragments, which activate stromal NF-κB signaling. This stromal activation further increases IL-6, S100A8/A9, and TGF-β, creating a feedback loop that selectively supports *TP53*-mutant progenitors. Macrophages in TP53-mutated disease exhibit impaired efferocytosis, promoting the accumulation of necrotic debris and inflammasome activation [87,90].

**Figure 5:**
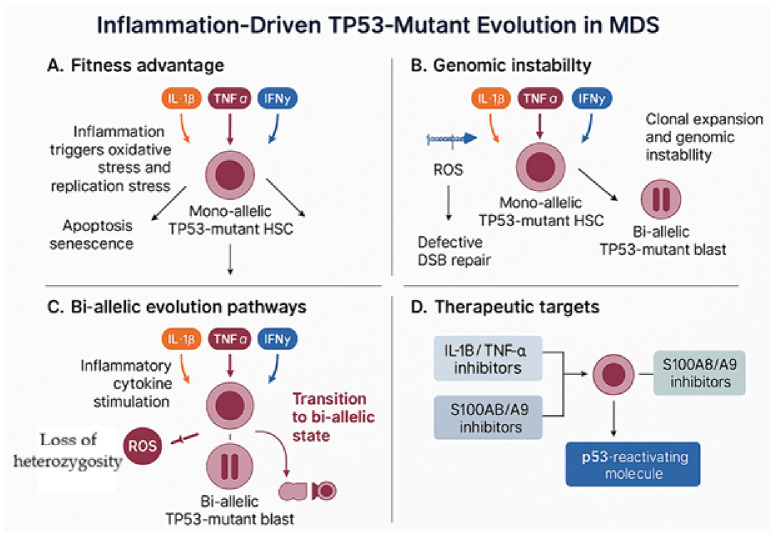
Inflammation-driven TP53-mutant evolution in MDS. (**A**) Fitness advantage: Pro-inflammatory cytokines impose oxidative and replicative stress on hematopoietic stem cells (HSCs), selectively favoring the survival and expansion of monoallelic *TP53*-mutant clones. (**B**) Genomic instability: Chronic inflammatory signaling increases oxidative stress and impairs DNA damage responses, leading to the accumulation of chromosomal aberrations and secondary mutations. (**C**) Biallelic evolution: Repeated inflammatory stress accelerates the transition to biallelic *TP53* inactivation through the loss of heterozygosity, secondary mutations, or chromosomal deletions, conferring maximal leukemogenic potential. (**D**) Therapeutic implications: Targeting inflammatory selection pressures and inflammation-associated pathways represents a strategy to limit *TP53*-mutant clonal fitness.

Finally, as will be addressed more comprehensively later in this review, the interplay between *TP53* mutations and inflammation suggests potential therapeutic strategies currently under clinical investigation:

- Targeting IL-1β/TNF-α to reduce inflammatory selection pressure;
- IRAK4 inhibition, relevant in spliceosome co-mutated *TP53* cases;
- Anti-CD47 therapy to restore macrophage-mediated clearance;
- *P53*-reactivating molecules for specific missense variants;
- Targeting S100A8/A9–TLR4 pathways, which are especially active in *TP53*-high clones (Figure 5).

Early-phase clinical studies targeting IRAK4 signaling, CD47 blockade, and inflammatory pathways have shown promising but still preliminary results, highlighting the need for further validation in larger trials [91].

Understanding inflammation-driven *TP53* clonal expansion provides a conceptual framework to design therapies that reduce inflammatory “fuel” sustaining these lethal clones [92].

## 8. Spliceosomal Dysfunction as a Driver of Inflammatory Signaling and Clonal Fitness in Myelodysplastic Syndromes

Spliceosome mutations—most notably involving *SF3B1*, *SRSF2*, *U2AF1*, and *ZRSR2*—represent one of the defining molecular hallmarks of MDS, collectively affecting more than half of all patients. These lesions alter pre-mRNA splicing fidelity, generating aberrant transcripts that disrupt gene regulation, cellular metabolism, and immune homeostasis. Increasing evidence shows that spliceosome-mutated MDS displays distinct inflammatory phenotypes, highlighting a mechanistic link between splicing dysregulation and inflammation-driven clonal evolution [93,94,95]. Notably, the inflammatory and transcriptional consequences of *SRSF2* mutations may differ depending on specific co-mutation patterns. In the presence of *SRSF2* and *EZH2* co-mutation alone, loss of PRC2 function leads to the partial derepression of inflammatory and myeloid differentiation programs, resulting in moderate cytokine upregulation and myeloid skewing [49]. In contrast, the additional co-mutation of *BCOR*, a component of the PRC1.1 complex, results in combined disruption of PRC1 and PRC2-mediated repression. This dual epigenetic impairment amplifies transcriptional deregulation and enhances the expression of inflammatory mediators such as CCL3, TNFα, and S100A8/A9, thereby reinforcing a stronger inflammatory phenotype and increased clonal fitness [49].

Aberrant splicing resulting from mutations in *SF3B1* and *U2AF1* leads to the accumulation of diverse categories of misprocessed RNA species, including nonsense-mediated decay substrates, circular RNAs, defective mitochondrial transcripts, and endogenous retroelement-derived RNAs. These nucleic acid species function as aberrant danger signals that engage cytosolic pattern-recognition receptors such as MDA5, RIG-I, and the cGAS–STING axis. Their activation leads to robust type I and type II interferon responses, promoting a sustained inflammatory milieu. The resulting cytokine signaling not only amplifies systemic inflammation but also suppresses erythropoiesis, contributing to the anemia that characterizes several spliceosome-mutated MDS subtypes [26,95,96,97].

A major conceptual advance has been the discovery that *U2AF1* mutations drive an alternative splicing event that favors expression of the hyperactive IRAK4-Long isoform. This aberrant form of IRAK4 strongly activates the MyD88–NF-κB pathway, creating a state of constitutive innate immune activation. In addition to IRAK4–NFκB-mediated signaling, *U2AF1* mutations have been linked to alternative inflammatory mechanisms involving transcriptional and metabolic reprogramming. In particular, *U2AF1*-mutant cells exhibit activation of the transcription factor FOXO3a, which promotes autophagy-related programs and contributes to disease progression. This pathway is mechanistically coupled with activation of the NLRP3 inflammasome, leading to the increased production of inflammatory mediators and induction of pyroptotic cell death. These findings further highlight how spliceosome mutations integrate with innate immune signaling pathways to amplify inflammation-driven clonal evolution in MDS [98].

Patients harboring IRAK4-Long-driven disease exhibit elevated levels of IL-8, IL-6, and TNF-α, and as reported above, preclinical models indicate that IRAK4 inhibition can restore more physiological hematopoiesis, underscoring the translational relevance of this pathway [99,100].

In *SF3B1*-mutant MDS, inflammation intersects mitochondrial dysfunction. Mis-splicing of genes required for mitochondrial heme metabolism results in ineffective erythropoiesis and accumulation of ring sideroblasts. Mitochondrial stress increases the production of ROS, which in turn activates inflammasome complexes and enhances the secretion of IL-1β and IL-18. Multi-omics factor analysis has linked these inflammatory signatures to broader aging-related pathways, suggesting that *SF3B1* mutations propagate a combined mitochondrial–inflammatory program [101,102].

Mutations in *SRSF2* further illustrate how spliceosome dysfunction promotes inflammatory myelopoiesis. Mis-splicing of key regulators of hematopoietic differentiation—such as *EZH2* and *BCOR*—drives a myeloid-skewed phenotype accompanied by high expression of *CCL3*, *TNF-α*, and *S100A8/A9* in granulocyte–macrophage progenitors. These inflammatory mediators create a self-reinforcing loop that restricts lymphoid output, enhances myeloid bias, and contributes to clinical severity [49,103].

**Figure 6:**
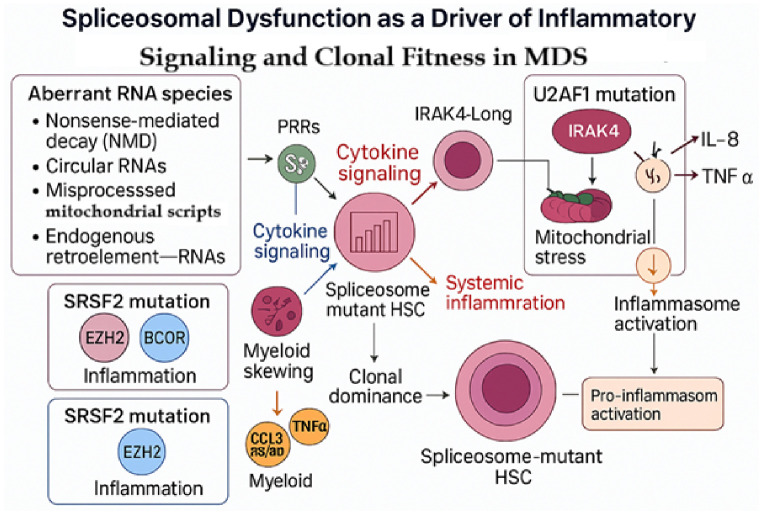
Spliceosome mutations generate aberrant RNA species that activate innate immune sensors, drive cytokine and inflammasome signaling, reshape the marrow niche, and promote clonal dominance of spliceosome-mutant HSCs. In *SRSF2*-mutant contexts, co-mutation patterns modulate the magnitude of inflammatory signaling: *SRSF2–EZH2* co-mutation primarily reflects PRC2 loss-driven myeloid skewing, whereas additional *BCOR* alterations further disrupt PRC1-mediated repression, amplifying inflammatory gene expression and clonal fitness.

Beyond cell-intrinsic mechanisms, spliceosome-mutated clones influence the bone marrow microenvironment. Misprocessed RNAs are released via exosomes and internalized by stromal cells, where they trigger NF-κB activation and promote the differentiation of stromal elements into a pro-inflammatory state. This remodeled niche preferentially supports mutated HSCs while suppressing normal hematopoiesis, thereby amplifying clonal dominance and contributing to disease heterogeneity [6] (Figure 6).

## 9. Autoimmune and Inflammatory Comorbidities in MDS: Clinical Significance and Distinct Phenotypic Patterns

Systemic inflammatory and autoimmune diseases (SIADs) represent a clinically relevant extra-hematologic manifestation in patients with MDS, with a reported prevalence of 20–30%. The clinical spectrum is broad and heterogeneous, reflecting the complex immune perturbations that accompany clonal myeloid disorders. Frequently observed SIADs include small- and medium-vessel vasculitis, seronegative polyarthritis, Behçet-like mucocutaneous involvement, neutrophilic dermatoses such as Sweet syndrome, autoimmune cytopenias, and various forms of thyroid autoimmunity. These disorders can be presented as isolated organ-specific inflammatory reactions or as systemic syndromes marked by fever, cutaneous eruptions, and elevated inflammatory markers.

Among the recently recognized entities, VEXAS (vacuoles, E1 enzyme, X-linked, autoinflammatory, somatic) syndrome represents a paradigmatic example of the intersection between somatic mutations and systemic inflammation. This syndrome is caused by acquired mutations in the *UBA1* gene in hematopoietic progenitor cells and is characterized by severe autoinflammatory manifestations, including fever, vasculitis, dermatologic involvement, and pulmonary inflammation. Importantly, a significant proportion of patients with VEXAS syndrome develop or fulfill diagnostic criteria for MDS or other clonal hematologic disorders.

From a pathogenic standpoint, VEXAS highlights how somatic mutations in hematopoietic stem cells can directly drive systemic immune dysregulation, supporting the concept that inflammatory and autoimmune manifestations in MDS may, at least in part, reflect the intrinsic biology of the malignant clone rather than secondary immune phenomena alone [104].

Notably, SIADs may arise before, at the time of, or after the diagnosis of MDS, underscoring the bidirectional relationship between immune dysregulation and clonal hematopoiesis [105,106,107,108].

The temporal dissociation between the onset of SIADs and the identification of dysplasia has led to particular interest in the role of immune activation as a sentinel of evolving marrow pathology. In a subset of patients, autoimmune manifestations precede the hematologic diagnosis by months or years, raising the possibility that abnormalities in immune regulation may serve as an early indicator of subclinical clonal expansion. Conversely, in patients with established MDS, SIADs often correlate with episodes of increased inflammatory drive rather than with overt disease progression, supporting the concept that they arise from qualitative immune dysfunction rather than quantitative bone marrow failure [109].

Cytogenetic–phenotypic correlations further illuminate the relationship between MDS and autoimmunity [102]. Trisomy 8 is strongly associated with Behçet-like manifestations, including recurrent oral and genital ulcers and gastrointestinal involvement [103,104,105]. This association has been attributed to the increased expression of pro-inflammatory pathways encoded on chromosome 8, which may sensitize tissues to mucosal inflammation [106,107].

Management of SIADs in the context of MDS typically involves immunomodulatory therapy—most commonly corticosteroids, methotrexate, or other conventional immunosuppressive agents—which often result in rapid symptomatic improvement. However, their influence on the hematologic course of MDS is variable. Disease-modifying treatments such as azacitidine have shown the capacity to ameliorate both SIAD manifestations and MDS-related cytopenias in a subset of patients through the suppression of aberrant cytokine production and the restoration of more balanced immune signaling. Nonetheless, autoimmune manifestations may remain refractory despite hematologic responses, highlighting the complexity of the underlying immune pathology [110].

Overall, SIADs in MDS should be interpreted as clinical expressions of the broader immune and inflammatory imbalance intrinsic to clonal myeloid disease, rather than as independent prognostic determinants.

In this context, the systemic inflammatory and immune dysregulation underlying SIADs is not confined to clinically overt manifestations but is also reflected in subtle and quantifiable alterations detectable in peripheral blood. Indeed, routine hematologic parameters may provide an accessible window into the same pathogenic processes that drive autoimmune phenomena, capturing the balance between inflammatory activation, immune exhaustion, and clonal hematopoiesis. Therefore, peripheral blood-derived inflammatory indices can be viewed as complementary tools that extend the clinical and biological insights derived from SIADs, offering a continuous and measurable representation of immune dysfunction in patients with MDS.

Peripheral blood-derived inflammatory indices provide a simple and clinically accessible surrogate of the immune and inflammatory landscape shaping MDS, capturing key aspects of innate and adaptive dysfunction, clonal dynamics, and marrow niche activity [111,112].

Among the single parameters, the absolute monocyte count (AMC) is particularly informative, as reduced levels reflect impaired innate immunity and defective efferocytosis, highlighting profound immune dysregulation beyond its prognostic value [113,114]. Similarly, the absolute lymphocyte count (ALC) serves as a marker of immune competence; lymphocytopenia (ALC < 1.2 × 10^9^/L) correlates with increased infection risk, diminished T-cell diversity, and impaired NK cell function. Importantly, the reduction in ALC in MDS is multifactorial and reflects both bone marrow failure and immune dysregulation. On the one hand, ineffective hematopoiesis limits lymphoid output from hematopoietic stem cells, contributing to quantitative lymphopenia. On the other, chronic antigenic stimulation and persistent inflammatory signaling drive immune exhaustion, particularly within T-cell compartments [115,116,117].

More specifically, CD8^+^ T cells frequently exhibit an exhausted phenotype characterized by reduced cytokine production and impaired cytotoxic function, while CD4^+^ T cells show altered helper responses and skewing toward regulatory or dysfunctional subsets. In parallel, B-cell lymphopenia and reduced receptor diversity further compromise humoral immunity. Together, these quantitative and qualitative alterations explain the decline in ALC and its association with increased susceptibility to infections, reduced immune surveillance, and poorer clinical outcomes in MDS.

Composite indices further refine this assessment. The neutrophil-to-lymphocyte ratio (NLR) integrates inflammatory myelopoiesis and lymphocyte suppression, with elevated values (>4–5) associated with adverse outcomes, including higher blast burden, increased IL-8 levels, and reduced response to hypomethylating agents. The platelet-to-lymphocyte ratio (PLR) reflects thrombopoietic stress and systemic inflammation and has been consistently linked to inferior event-free survival [12,111,118,119]. The monocyte-to-lymphocyte ratio (MLR), often increased in advanced disease, correlates with myeloid expansion and disease progression, capturing early shifts toward inflammatory clonal evolution even in lower-risk MDS [120,121].

Collectively, these indices provide an integrated and dynamic snapshot of systemic inflammation and immune competence. While individually modest, their combined interpretation complements genetic and cytogenetic data and supports their incorporation into refined prognostic models and therapeutic decision-making frameworks (Table 2).

## 10. Inflammation-Targeted Therapy in MDS: A Conceptual and Therapeutic Shift

Targeting inflammation has emerged as one of the most promising and conceptually transformative directions in the management of MDS. As understanding of MDS pathobiology has evolved, it has become increasingly evident that inflammatory signaling is not simply an epiphenomenon of marrow dysfunction, but a central driver of key disease features—including ineffective hematopoiesis, clonal selection, bone marrow microenvironment remodeling, and immune dysregulation. Therapeutic strategies aimed at modulating these inflammatory circuits therefore represent a rational and biologically grounded complement to cytotoxic and epigenetic therapies, with the potential to alter both symptom burden and disease trajectory [13].

For the purposes of this review, the terms “early-stage” and “advanced-stage” MDS broadly correspond to lower-risk and higher-risk disease categories according to established prognostic scoring systems, including IPSS-R and IPSS-M. Since inflammatory and immune signatures largely correlate with biological risk rather than chronological disease duration, the terminology lower-risk and higher-risk MDS may be considered more appropriate in a clinical context.

### 10.1. Therapeutic Strategies in Lower-Risk MDS

In early-stage MDS, the immune microenvironment is predominantly characterized by heightened inflammatory signaling and immune activation. In this context, excessive innate immune activation contributes to ineffective hematopoiesis and increased apoptosis. Therefore, therapeutic strategies aimed at reducing inflammation and restoring hematopoietic function may be beneficial.

Approaches under investigation include the inhibition of innate immune pathways, such as targeting NLRP3 inflammasome activation, IL-1 signaling, and the TLR pathways, as well as the use of hypomethylating agents that can modulate inflammatory signaling. Additionally, therapies aimed at restoring immune balance without excessive immunosuppression are of particular interest in this disease stage.

Among the most extensively investigated inflammatory pathways is the IL-1β axis, which orchestrates pyroptotic cell death and early hematopoietic failure in MDS. Excessive IL-1β signaling promotes inflammasome activation and skews hematopoietic progenitors toward apoptosis, particularly within the erythroid compartment. Pharmacologic inhibition of IL-1β or its receptor—via agents such as canakinumab and anakinra—or upstream suppression of NLRP3 inflammasome activity might attenuate inflammatory apoptosis and restore erythropoietic output. These observations support ongoing efforts to evaluate IL-1β blockade in MDS patients with prominent inflammatory signatures [31,49].

Clinical evidence further supports the relevance of IL-1β signaling in MDS. A phase II clinical trial investigating the IL-1β inhibitor canakinumab in patients with previously treated lower-risk MDS demonstrated the feasibility of targeting this pathway, with evidence of biological and clinical activity in a subset of patients. In addition, elevated IL-1β levels and associated alterations in regulatory T-cell subsets have been correlated with disease progression, further supporting the pathogenic role of this pathway [122,123].

Another major inflammatory pathway relevant to MDS pathogenesis involves TGF-β, a cytokine that enforces erythroid maturation arrest, promotes stromal fibrosis, and contributes to immunosuppressive niche formation.

Luspatercept, a ligand trap for TGF-β superfamily members, improves erythropoiesis and reduces transfusion burden in lower-risk MDS, particularly in *SF3B1*-mutant disease. Similarly, sotatercept acts through the modulation of TGF-β signaling to restore late-stage erythroid maturation. In addition, galunisertib (LY2157299), a TGF-β receptor I kinase inhibitor, represents a promising investigational strategy aimed at disrupting this pathway at the signaling level. These approaches highlight the central role of TGF-β signaling in MDS and its therapeutic exploitation [124,125].

Modulation of TGF-β superfamily signaling through agents such as luspatercept has already transformed treatment for SF3B1-mutant and ring sideroblast-associated MDS, where the restoration of erythroid maturation has yielded clinically meaningful reductions in transfusion burden. Efforts are underway to expand TGF-β-directed therapies to broader MDS subsets, given the pervasive role of this pathway in hematopoietic regulation and fibrosis [32,82,126,127].

Inflammatory pathways linked to innate immune sensors also present actionable therapeutic vulnerabilities. IRAK4, a key kinase downstream of Toll-like receptors and MyD88, is aberrantly activated in certain spliceosome-mutant MDS, particularly those harboring *U2AF1* mutations that generate the hyperactive IRAK4-Long isoform. Inhibitors of IRAK4, such as emavusertib, have demonstrated potent suppression of NF-κB-driven inflammatory transcriptional programs and have shown encouraging preclinical activity, especially in combination with hypomethylating agents [13,99,100,128].

Targeting damage-associated inflammatory signals have similarly gained traction. The alarmins S100A8 and S100A9—potent activators of TLR4 and NLRP3 inflammasomes—are enriched in high-risk MDS, del(5q) disease, and TP53-mutated clones. Neutralizing antibodies and small-molecule inhibitors directed against S100A8/A9 aim to interrupt this pathological loop of innate immune priming, inflammatory cell death, and erythroid suppression [13,129].

### 10.2. Therapeutic Strategies in Higher-Risk MDS

In higher-risk MDS, disease progression is characterized by increasing immune exhaustion, immune escape mechanisms, and the expansion of aggressive clones. Although current standard treatment is based on hypomethylating agents, while allogeneic hematopoietic stem cell transplantation remains the only potentially curative option for eligible patients, in this setting, immune-modulatory therapies are being investigated as complementary strategies aimed at improving treatment response and overcoming immune dysfunction.

Immune checkpoint inhibitors targeting the PD-1/PD-L1 and TIM-3 pathways represent a key area of investigation, often in combination with hypomethylating agents. Additional approaches include CD47 blockade to enhance macrophage-mediated phagocytosis, as well as strategies targeting the tumor-supportive microenvironment. These therapeutic paradigms reflect the shift from a pro-inflammatory to an immune-evasive disease state during MDS progression.

Although traditionally viewed as epigenetic therapies, hypomethylating agents (HMAs) such as azacitidine and decitabine possess substantial immunomodulatory properties. They reduce pro-inflammatory cytokines, enhance antigen presentation, reprogram exhausted T cells, and promote regulatory T cell expansion. Clinical response to HMAs often correlates with a decline in inflammatory gene expression, underscoring the interplay between epigenetic and immunologic mechanisms [130,131,132].

Another emerging class of therapies includes agents that interfere with the “don’t-eat-me” signal mediated by CD47-SIRPα, a pathway exploited by dysplastic clones to evade macrophage-mediated phagocytosis. Anti-CD47 therapies restore innate immune clearance and may synergize with anti-inflammatory agents by simultaneously reducing clonal fitness and dampening inflammatory feedback loops [133,134].

Finally, senolytic therapies—designed to eliminate senescent stromal and immune cells—represent an innovative approach to dismantling the chronically inflamed marrow niche characteristic of MDS. By selectively targeting these senescent cellular populations, senolytics aim to reverse maladaptive microenvironmental remodeling and restore a more permissive environment for normal hematopoiesis [135,136].

Given the multifaceted ways in which inflammation shapes disease biology, future treatment paradigms will involve strategic combinations of anti-inflammatory agents, hypomethylating therapies, metabolic modulators, immune checkpoint inhibitors, and targeted anti-clonal drugs. Such integrated approaches aim not only to mitigate inflammatory selection pressures, but also to enhance immune surveillance, reduce clonal advantage, and reestablish bone marrow homeostasis [13] (Table 3).

However, although several inflammation-targeted approaches have shown encouraging biological and clinical activity in early-phase studies, most remain investigational. Larger prospective studies and randomized clinical trials are required to validate their efficacy, define optimal patient selection strategies, and establish their role within current therapeutic algorithms for MDS.

## 11. Conclusions

MDS emerge at the crossroads of genetic mutations, chronic inflammation, and bone marrow niche dysfunction. Inflammaging shapes HSC vulnerability; innate and adaptive immune dysregulation perpetuate malignant selection; and the marrow microenvironment becomes progressively reprogrammed to favor clonal expansion. Mutations in TP53, splicing factors, and epigenetic regulators interact dynamically with cytokine networks, creating disease-specific inflammatory phenotypes.

Clinically, inflammatory biomarkers provide accessible tools for risk refinement, while SIADs highlight shared pathogenic pathways. Therapeutically, the recognition of MDS as an inflammatory disease has opened new avenues through IL-1β, IRAK4, TGF-β, and S100A8/A9 targeting strategies. The future of MDS therapy lies in the precision modulation of inflammation, integrated with genomic and microenvironmental profiling.

## Figures and Tables

**Table 1:** Principal inflammatory pathways contributing to MDS initiation and progression, integrating innate immune activation, cytokine-driven signaling, inflammasome activity, and stromal inflammatory remodeling.

Pathway/Component	Mechanistic Role in MDS	Downstream Effects	Representative Mutations/Contexts
TLR–MyD88–IRAK4 axis	Chronic activation of innate immune receptors; sustained inflammatory cytokine production	NF-κB activation; IL6/IL8/TNFα overexpression; impaired hematopoiesis	*SRSF2, U2AF1 (IRAK4-Long isoform)*
NLRP3 inflammasome	Caspase-1 activation and IL1β/IL18 maturation	Pyroptosis; intramedullary apoptosis; ROS accumulation	Lower-risk MDS
S100A8/A9–TLR4 signaling	DAMP-mediated chronic inflammation	Oxidative stress; erythroid suppression; inflammasome priming	del(5q) MDS; *TP53*-mutant MDS
TNFα/IL1β cytokine stress	Suppression of normal HSC quiescence; inflammatory selection pressure	Myeloid skewing; HSC exhaustion; survival of resistant clones	*TET2, ASXL1, TP53*
Interferon (IFN-I/II) signaling	Activation by mis-spliced RNA or retroelements	Adaptive immune exhaustion; impaired erythropoiesis	*SF3B1, U2AF1*
*NF-κB* hyperactivation	Persistent transcription of inflammatory programs	IL6/IL8 overexpression; stromal activation; erythroid suppression	*U2AF1 (IRAK4* *-* *Long)*
TGF-β signaling	Suppression of erythroid maturation	Ineffective erythropoiesis; niche fibrosis	*SF3B1* *-* *mutant disease*
SASP phenotype	Release of IL6, IL8, IL1β from senescent cells	Myeloid skewing; metabolic dysfunction; inflammaging	*Aging-associated MDS; CHIP carriers*

**Table 2:** Key inflammatory and immune-derived hematologic parameters associated with MDS pathobiology and prognosis.

Biomarker	Pathophysiologic Interpretation	Associated Clinical Outcomes	Refs.
Absolute monocyte count (AMC)	Expansion of pro-inflammatory monocytes	Worse survival; higher risk of AML progression	[113,114]
Absolute lymphocyte count (ALC)	Lymphopenia reflecting immune exhaustion	Poorer survival; inferior response to HMAs	[115,116,117]
Neutrophil-to-lymphocyte ratio (NLR)	Indicator of systemic inflammation	Predictor of adverse prognosis	[12,111,118,119]
Platelet-to-lymphocyte ratio (PLR)	Platelet-mediated inflammatory activity	Associated with transfusion dependence	[12,111,118,119]
Monocyte-to-lymphocyte ratio (MLR)	Balance between inflammatory and regulatory compartments	Correlates with high-risk MDS states	[120,121]
Cytokine levels (IL6, TNFα, IL1β)	Drivers of apoptosis and ineffective hematopoiesis	Correlate with disease severity and therapy response	[87,90]
S100A8/A9	DAMP signaling and inflammasome activation	Elevated in del(5q) and TP53-mutant MDS	[52,53,54]
CRP/inflammatory indices	Reflect systemic inflammatory status	Predict poorer HMA response	[12]

**Table 3:** Immunomodulatory therapeutic strategies in MDS according to disease stage.

Target/Pathway	Drug/Strategy	Mechanism of Action	Disease Stage	Development Stage
NLRP3 inflammasome	NLRP3 inhibitors	Inhibition of inflammasome activation	Lower-risk MDS	Preclinical
IL-1 signaling	Anakinra	IL-1 receptor blockade	Lower-risk MDS	Clinical (early-phase)
TLR signaling	IRAK4 inhibitors	Block innate immune activation	Lower-risk MDS	Clinical
DNA methylation	Azacitidine/Decitabine	Epigenetic and immune modulation	Both	Approved
PD-1/PD-L1	Nivolumab, Pembrolizumab	Restore T-cell activity	Higher-Risk MDS	Clinical trials
TIM-3	Sabatolimab	Reverse T-cell exhaustion	Higher-Risk MDS	Clinical trials
CD47	Magrolimab	Enhance macrophage phagocytosis	Higher-Risk MDS	Clinical trials
Treg/microenvironment	Experimental agents	Modulate immunosuppressive niche	Higher-Risk MDS	Preclinical/Clinical

## Data Availability

No new data were created or analyzed in this study.

## Abbreviations

The following abbreviations are used in this manuscript:

Abbreviation	Full Term
ALC	Absolute Lymphocyte Count
AML	Acute Myeloid Leukemia
AMC	Absolute Monocyte Count
BME	Bone Marrow Microenvironment
CCUS	Clonal Cytopenia of Undetermined Significance
cGAS	Cyclic GMP–AMP Synthase
CHIP	Clonal Hematopoiesis of Indeterminate Potential
CRP	C-Reactive Protein
DAMPs	Damage-Associated Molecular Patterns
DCs	Dendritic Cells
ECM	Extracellular Matrix
ERVs	Endogenous Retroviruses
GM-CSF	Granulocyte–Macrophage-Colony-Stimulating Factor
HMAs	Hypomethylating Agents
HSCs	Hematopoietic Stem Cells
IFN	Interferon
IPSS	International Prognostic Scoring System
IRAK	Interleukin-1 Receptor-Associated Kinase
LOH	Loss of Heterozygosity
MAPK	Mitogen-Activated Protein Kinase
MDA5	Melanoma Differentiation-Associated Protein 5
MDS	Myelodysplastic Syndromes
MLR	Monocyte-to-Lymphocyte Ratio
MSCs	Mesenchymal Stromal Cells
MyD88	Myeloid Differentiation Primary Response 88
NF-κB	Nuclear Factor Kappa-Light-Chain-Enhancer of Activated B Cells
NLRP3	NACHT, LRR, and PYD Domains-Containing Protein 3
NK	Natural Killer
NLR	Neutrophil-to-Lymphocyte Ratio
PLR	Platelet-to-Lymphocyte Ratio
PRR	Pattern Recognition Receptor
ROS	Reactive Oxygen Species
RIG-I	Retinoic Acid-Inducible Gene I
SASP	Senescence-Associated Secretory Phenotype
SIADs	Systemic Inflammatory and Autoimmune Diseases
STING	Stimulator of Interferon Genes
TGF-β	Transforming Growth Factor Beta
TLR	Toll-Like Receptor
TNF-α	Tumor Necrosis Factor Alpha
Tregs	Regulatory T Cells
